# Plant growth–promoting rhizobacteria: *Peribacillus frigoritolerans* 2RO30 and *Pseudomonas sivasensis* 2RO45 for their effect on canola growth under controlled as well as natural conditions

**DOI:** 10.3389/fpls.2023.1233237

**Published:** 2024-01-08

**Authors:** Joanna Świątczak, Agnieszka Kalwasińska, Maria Swiontek Brzezinska

**Affiliations:** Department of Environmental Microbiology and Biotechnology, Nicolaus Copernicus University in Toruń, Toruń, Poland

**Keywords:** PGPR, canola, sterile and non-sterile conditions, genome analysis, *Peribacillus frigoritolerans*, *Pseudomonas sivasensis*

## Abstract

Even though canola is one of the most important industrial crops worldwide, it has high nutrient requirements and is susceptible to pests and diseases. Therefore, natural methods are sought to support the development of these plants. One of those methods could be a plant growth–promoting rhizobacteria (PGPR) that have a beneficial effect on plant development. The aim of this study was a genomic comparison of two PGPR strains chosen based on their effect on canola growth: *Peribacillus frigoritolerans* 2RO30, which stimulated canola growth only in sterile conditions, and *Pseudomonas sivasensis* 2RO45, which promoted canola growth in both sterile and non-sterile conditions. First of all, six bacterial strains: RO33 (*Pseudomonas* sp.), RO37 (*Pseudomonas poae*), RO45 (*Pseudomonas kairouanensis*), 2RO30 (*Peribacillus frigoritolerans*), 2RO45 (*Pseudomonas sivasensis*), and 3RO30 (*Pseudomonas migulae*), demonstrating best PGP traits *in vitro*, were studied for their stimulating effect on canola growth under sterile conditions. *P. frigoritolerans* 2RO30 and *P. sivasensis* 2RO45 showed the best promoting effect, significantly improving chlorophyll content index (CCI) and roots length compared to the non-inoculated control and to other inoculated seedlings. Under non-sterile conditions, only *P. sivasensis* 2RO45 promoted the canola growth, significantly increasing CCI compared to the untreated control and to other inoculants. Genome comparison revealed that the genome of *P. sivasensis* 2RO45 was enriched with additional genes responsible for ACC deaminase (*acdA*), IAA (*trpF*, *trpG*), and siderophores production (*fbpA*, *mbtH*, and *acrB*) compared to 2RO30. Moreover, *P. sivasensis* 2RO45 showed antifungal effect against all the tested phytopathogens and harbored six more biosynthetic gene clusters (BGC), namely, syringomycin, pyoverdin, viscosin, arylpolyene, lankacidin C, and enterobactin, than *P. frigoritolerans* 2RO30. These BGCs are well known as antifungal agents; therefore, it can be assumed that these BGCs were responsible for the antifungal activity of *P. sivasensis* 2RO45 against all plant pathogens. This study is the first report describing *P. sivasensis* 2RO45 as a canola growth promoter, both under controlled and natural conditions, thus suggesting its application in improving canola yield, by improving nutrient availability, enhancing stress tolerance, and reducing environmental impact of farming practices.

## 1 Introduction

Plant growth–promoting rhizobacteria (PGPR) that colonize plant rhizosphere are group of bacteria exerting a beneficial effect on plant development (Adedeji et al., 2020). The application of these microorganisms can reduce the requirement of synthetic chemical fertilizers and pesticides, which is important for the development of sustainable agriculture (Patel et al., 2021). PGPR can promote plant growth directly or indirectly. Direct plant growth promotion includes mechanisms providing plants with phosphorus, nitrogen, iron, and indol-3-acetic-acid, while indirect promotion consists of preventing phytopathogens due to antimicrobial metabolites and extracellular enzymes (Chouyia et al., 2020; Wang et al., 2023). Many beneficial microorganisms, including certain bacteria and fungi, produce antibiotics as secondary metabolites, inhibiting the growth and development of phytopathogens by disrupting their cell walls, membranes, or metabolic processes. Beneficial microorganisms can outcompete phytopathogens for essential nutrients in the rhizosphere. Additionally, some microbial metabolites can induce systemic resistance in plants, making them more resistant to pathogenic attacks. By breaking down the cell walls of phytopathogens, bacterial enzymes such as chitinases and glucanases inhibit the growth and spread of pathogens. Meanwhile, extracellular proteases and lipases produced by beneficial microorganisms can hydrolyze proteins and lipids in the cell membranes (Kumari et al., 2019; de Andrade et al., 2023).

The genus *Pseudomonas* and *Bacillus* are well known as PGPRs and biocontrol agents due to their ability to solubilize phosphates and produce phytohormones or secondary metabolites such as hydrogen cyanide (HCN), siderophores, and lipopeptides (Sheoran et al., 2015; Lastochkina et al., 2019; Oni et al., 2022). It was reported that bio-inoculations of the three PGPR *Bacillus* strains with the ability to catabolize ACC, increased shoots, and roots length of canola plants (Kashyap et al., 2019). Whereas *Pseudomonas* sp. strain, which was able to produce siderophores and fix nitrogen, affected canola seeds germination and seedlings growth (Jamalzadeh et al., 2021). Ecological applications of *Pseudomonas* and *Bacillus* species as bioinoculants are crucial for maintaining food security (Sah et al., 2021; Etesami et al., 2023).

*Peribacillus frigoritolerans* is a rod-shaped, Gram-positive bacterium belonging to the family *Bacillaceae* and classified originally as *Brevibacterium frigoritolerans* (Montecillo and Bae, 2022). It is well documented that *Brevibacterium frigoritolerans* stimulates plant growth and suppresses diseases caused by phytopathogens. *B. frigoritolerans* promotes growth of wheat (*Triticum aestivum* L.) increasing roots, shoots, seedlings length, and plant biomass (Tara and Saharan, 2017), while Raufa et al. (2019) found this strain as biocontrol agent in suppression of maize (*Zea mays* L.) stalk rot caused by *Fusarium moniliforme*. Whereas, *Pseudomonas sivasensis* was isolated from farm fisheries in Turkey and described for the first time by Duman et al. (2020). Świątczak et al. (2023a) found that PGPR *Pseudomonas sivasensis* bacterization altered the taxonomic structure of bacterial and fungal communities by increasing the abundance of plant beneficial microorganisms and increasing metabolic activity and functional diversity of microbial communities in the canola rhizosphere.

In our study, we present new insights into the plant growth–promoting (PGP) properties of *Pseudomonas sivasensis* 2RO45, which promoted canola growth under both controlled and natural conditions. Moreover, PGP and biosynthetic cluster genes of *Pseudomonas sivasensis* 2RO45 were compared with other PGPR—*Peribacillus frigoritolerans* 2RO30 that promoted canola growth only in sterile conditions.

## 2 Materials and methods

### 2.1 Isolation of plant growth–promoting rhizobacteria

Canola (*Brassica napus* L. var. *napus*) plant samples collected from three following growth stages: vegetative (RO), flowering (2RO), and maturity (3RO) were sourced from farmland in Ostroda, Poland (53°41′38″N 19°57′58″E). To isolate bacteria from the rhizosphere, canola roots were washed with sterile distilled water and cut into small pieces (2 mm). Following the serial dilution process, each 10-fold diluent was spread onto nutrient agar (NA) (Biomaxima, Warsaw, Poland) plates with amphotericin B (40 µg/mL) as an antifungal agent and incubated for 3 days at 28°C. After incubation, the rhizospheric bacteria were enumerated and 50 colonies from each plant growth stage were isolated and stored in glycerol stocks at −80°C until further studies.

### 2.2 Assay of plant growth–promoting traits *in vitro*

#### 2.2.1 Qualitative estimations

The rhizobacterial strains were qualitatively screened for PGP traits, namely, phosphates, siderophores, chitinases, hydrogen cyanide (HCN), and ammonia production. Phosphorus solubilization and siderophores sequestration abilities were assessed using Pikovskaya agar plates and Chrome Azurol Sulphonate (CAS) medium, respectively (Pikovskaya, 1948; Alexander and Zuberer, 1991). Pikovskaya agar plates were incubated at 26°C for 7 days for observation of a halo zone around the colonies. The solubilization index (SoI) was evaluated as the ratio of total diameter (colony + halo zone) to colony diameter (Luziatelli et al., 2019). The CAS plates were incubated for 4 days at 26°C for observation of an orange halo zone around the colonies. The siderophores index (SI) was calculated as the ratio of the halo zone diameter to colony diameter. For chitinase production, bacteria were inoculated on medium containing (g/L): peptone 1.0, FeSO_4_ × 7H_2_O 0.1, iron gluconate 0.1, yeast extract 0.1, colloidal chitin 7.0 g dry mass, and agar 15.0 (Swiontek Brzezinska et al., 2013). Plates were incubated for 14 days at 22°C to observe clearing zone around the colonies. The colloidal chitin was prepared with the Lingappa and Lockwood method (1962). HCN production was checked using NA plates supplemented with glycine (0.44%) according to Lorck (1948). A sterile filter paper (Whatman No. 1) was soaked in solution of picric acid (0.5%) and sodium carbonate (2%) and was kept on the plate lid. After 4 days of incubation at 28°C, color change of filter paper from yellow to brown was a positive indicator for HCN production. Ammonia production was assessed by inoculating bacteria in nutrient broth (Biomaxima, Warsaw, Poland) for 3 days at 26°C. After adding a Nessler reagent (0.5 mL), the development of orange color was considered as a positive result.

#### 2.2.2 Quantitative estimations

##### 2.2.2.1 Indole acetic acid production

Bacterial indole acetic acid (IAA) may increase roots length and surface, allowing the plant better uptake of soil nutrients and water, which, in turn, can stimulate plant growth (Gilbert et al., 2018).

IAA production was quantified using medium (g/L): peptone 5.0; yeast extract 3.0; and L-tryptophan 1.0 following the modified method of Ehmann (1977). After 4 days of incubation at 28°C, culture suspension was centrifuged at 10080*g* for 10 min. Centrifuged culture suspension supernatant was mixed with Salkowski reagent (2% 0.5 FeCl_3_ in 35% HClO_4_) and incubated for 30 min in the dark. Intensity of the color was measured at 530 nm using Hitachi U-2500 spectrophotometer.

##### 2.2.2.2 ACC deaminase activity

PGP bacteria that express ACC deaminase activity may increase roots length and facilitate adaptation and survival of plants (del Carmen Orozco-Mosqueda et al., 2020).

Quantitative estimation of ACC was carried out according to the modified method described by Honma and Shimomura (1978). Bacterial cultures were inoculated in nutrient broth medium (Biomaxima, Warsaw, Poland), following incubation at 30°C for 24h in a rotary shaker. Culture suspension was centrifuged at 6000*g* for 10 min (4°C) and Dworkin and Foster (DF) salts minimal medium (5 mL) was added to the pellet (Dworkin and Foster, 1958). Culture suspension was centrifuged again at 6000*g* for 10 min (4°C) and DF salts minimal medium (5 mL) with 0.5 M ACC (30 μL) were added to the pellet. After incubation at 30°C for 24h, culture suspension was centrifuged at 4032*g* for 10 min (4°C) and bacterial cells were washed with 0.1M Tris-HCl (5 mL; pH 7.6). After centrifugation at 10000*g* for 5 min, 0.1 M Tris-HCl (600 μL; pH 8.5), toluene (30 μL), and 0.5 M ACC substrate (20 μL) were added to the pellet following incubation at 30°C for 30 min. 0.56 M HCl (1 ml) was added and culture suspension was centrifuged at 10000*g* for 5 min. Centrifuged culture suspension supernatant (1 mL) was mixed with 0.56 M HCl (800 μL) and 0.2% 2,4-dinitrophenylhydrazine (300 μL). After a 30-min long incubation at 30°C, 2 M NaCl (2 mL) was added and the absorbance was determined at 540 nm using Hitachi U-2500 spectrophotometer. The ACC deaminase was expressed in terms of nanomoles of α-ketobutyrate produced per milligram protein per hour. Protein content was estimated according Bradford (1976) method.

### 2.3 Greenhouse experiment in sterile and non-steriled conditions

#### 2.3.1 Seed sterilization and inoculum preparation

The winter rapeseed of the Areti variety was selected for the study. It is a hybrid variety characterized by outstanding health and a very high yield potential. Sterilization of canola seeds was performed by disinfection in 1% NaOCl for 30 min and by washing 3 times in sterile distilled water following the method of Rudolph et al. (2015). Fresh bacterial cultures were inoculated in LB broth and incubated for 2 days at 26°C in a shaker incubator. The sterilized seeds were resuspended in bacterial suspension (10 mL of 10^8^ CFU/mL) supplemented with 0.05 g carboxymethyl cellulose (CMC) and agitated for 30 min. The control was the seeds resuspended in 10 mL of nutrient broth without bacterial inoculum and supplemented with 0.5% CMC (Rudolph et al., 2015).

#### 2.3.2 Seed treatment

Four canola seeds were sown per pot in eight replicates for each treatment. Seeds resuspended in six bacterial suspension (RO33, RO37, RO45, 2RO30, 2RO45, and 3RO30) were germinated in a sterile sand and vermiculite (1:1). Seeds with 2RO30, 2RO45 strains and their consortium were additionally sown in non-sterile soil taken from the field in Ostroda, Poland (53°41′38″N 19°57′58″E). Canola seedlings were maintained in a day–night cycle of 16h light (100 μmol/m2/s) and a temperature of 22°C. The plants were moistened with an equal amount of water and were harvested after 44 days (Świątczak et al., 2023b).

#### 2.3.3 Compatibility assay

For non-sterile greenhouse experiment, *in-vitro* antagonism compatibility test of two strains: *Peribacillus frigoritolerans* 2RO30 and *Pseudomonas sivasensis* 2RO45 was performed. Each isolates were resuspended in sterile water (10^8^ CFU/mL) and 100 µL of the tested microorganism were spread onto NA plates. A sterile filter paper disc (5 mm in diameter) was placed onto NA plate containing the spread bacteria and 10 µL of bacterial suspension was inoculated on the paper disc. Plates were incubated at 28°C for 48h. When a clear zone of inhibition around the disc was observed, microorganisms were considered incompatible. When inhibition zone was not observed, they were classified as compatible (Widyantoro, 2019; Tabacchioni et al., 2021). Each experiment was performed in triplicate.

#### 2.3.4 Canola parameters determination

Chlorophyll content index (CCI) was measured using a chlorophyll meter CCM-200plus (Opti-Sciences, Hudson, USA). Other canola growth parameters – length of roots, shoots and epicotyl were measured after washing the plant roots with distilled water. Photosynthetic area of leaves was calculated using DigiShape 1.3 software (Moraczewski, 2005). The plant roots, shoots, epicotyl, petioles and leaves were dried at 85°C for 48 hours and dry weight of these plant parts was determined. Furthermore, two following indexes were calculated: specific leaf area (SLA) and leaf weight ratio (LWR). The SLA index was calculated as follows: SLA = assimilation area [cm^2^]/leaves dry biomass [g], while LWR index was evaluated as follows: LWR = leaves dry biomass [g]/total plant dry biomass [g] (Piernik et al., 2017).

#### 2.3.5 Soil physicochemical analyses

The physical and chemical parameters of the non-sterile soil were determined at District Chemical and Agricultural Station (Bydgoszcz, Poland). The physicochemical analyses such as pH, phosphorus, potassium, magnesium, ammonium nitrogen, nitrate nitrogen, and organic carbon were evaluated according to PN-ISO10390, 1997; PN-R-04020:1994/Az1, 2004; PN-R-04022, 1996; PN-R-04022:1996/Az1, 2002; PN-R-04028, 1997, and PN-ISO14235, 2003 standards, respectively.

### 2.4 Statistical analysis

The statistical analysis of the data was performed using Past3 (version 3.25). To determine significant differences between treatments, one-way analysis of variance (ANOVA) was applied, followed by Tuckey post-hoc test. ANOVA assumptions were checked using Shapiro–Wilk test for normality and Levene’s test for homogeneity of variances. When not normal distribution occurred, test for equal medians—Mann–Whitney was performed.

### 2.5 Identification of isolates based on 16S rRNA gene

Six bacterial isolates were identified based on 16S rRNA gene sequence according to Kalwasińska et al. (2020). Total genomic DNA was extracted using GeneMATRIX Bacterial and Yeast Genomic DNA Purification Kit (EURx, Gdańsk, Poland), following the manufacturer’s protocol. For polymerase chain reaction (PCR) amplification, the following 20 µL of reaction total volume was used: Taq DNA polymerase, 0.2 mM dNTP mixture, Polbuffer B with 1.5 mM MgCl_2_, 0.25µM of 27 F (5-AGAGTTTGATCCTGGCTCAG-3) and 1492 R (5-TACGGTTACCTTGTTACGACTT-3) primers, and 1 µL of genomic DNA. The PCR conditions were: initial denaturation (95°C for 3 min), 30 cycles of amplification: denaturation (95°C for 30s), annealing (52°C for 20s), extension (72°C for 1 min 40s) and final extension (72°C for 5 min). PCR amplicons were checked in 1% (w/v) agarose gel stained with Midori Green DNA Stain. Sequencing of PCR products was performed using Big Dye Terminator v 3.1 Cycle Sequencing Kit according to manufacturer’s instruction. Capillary electrophoresis was performed by the Sequencing and Oligonucleotides Synthesis Laboratory, IBB (Warsaw, Poland). Nucleotide sequences of RO33, RO37, RO45, 2RO30, 2RO45, and 3RO30 were submitted to GenBank under the accession numbers MW599360, MW599361, MW599362, MW599363, MW599366, and MW599367, respectively. The taxonomy of bacterial isolates were determined using the EzBioCloud database (Yoon et al., 2017).

### 2.6 Genome sequencing, prediction of PGP genes and biosynthetic gene cluster

Genomic DNA of *Peribacillus frigoritolerans* 2RO30 and *Pseudomonas sivasensis* 2RO45 was extracted and whole genome was sequenced on Illumina HiSeq using 250 bp paired-end protocol at the University of Birmingham (UK). Library construction was prepared with the use of Nextera XT Library Prep kit (Illumina, San Diego, CA, USA) according to the manufacturer’s instructions with the following modifications: PCR elongation time was increased from 30 s to 1 min, and 2 ng of DNA was used as an input instead of 1 ng. The library preparation and quantification of DNA were done with a Microlab STAR automated liquid handling system (Hamilton, Bonaduz, Switzerland). Quantification of pooled libraries was performed using the Kapa Biosystems Library Quantification Kit for Illumina on a Roche light cycler 96 qPCR machine. Reads were adapter trimmed using Trimmomatic 0.30 with a sliding window quality cutoff of Q15 (Bolger et al., 2014). *De-novo* assembly was performed on samples using SPAdes version 3.7 (Bankevich et al., 2012), and contigs were annotated using Prokka 1.11 (Seemann, 2014). Further annotation was performed using NCBI’s Prokaryotic Genome Annotation Pipeline (PGAP). The genomic sequences are available in DDBJ/ENA/GenBank under the accession numbers JAOAQM000000000 and JAOAQN000000000, respectively, for *Peribacillus frigoritolerans* 2RO30 and *Pseudomonas sivasensis* 2RO45.

The prediction of PGP genes was obtained from KEGG pathway analysis (https://www.kegg.jp/kegg/mapper/reconstruct.html), while secondary metabolite biosynthesis gene clusters were determined by antiSMASH (version 6.0) (https://antismash.secondarymetabolites.org).

### 2.7 Assay of plant pathogens growth inhibition *in vitro*

Bacterial isolates were screened for inhibition against fungal pathogens: *Alternaria alternata* 783, *Botrytis cinerea* 873, *Fusarium culmorum* 2333, *Fusarium oxysporum* 872, *Fusarium solani* 25, *Phytophthora cactorum* 1925, and *Phytophthora megasperma* 404, which can affect canola plants (Fernandez, 2007; Krasnow and Hausbeck, 2015; Al-Lami et al., 2019; Romero et al., 2008; Monnier et al., 2018; Ismaiel et al., 2021; Yu et al., 2023). Plant pathogenic fungi were obtained from the Plant Pathogenic Bank of the Institute of Plant Protection in Poznan (Poland). Fungi were cultivated on PDA plates (Biomaxima, Warsaw, Poland) for 5 days at 26°C, while bacteria were cultivated on PDA for 24h at 26°C. After incubation, mycelium agar discs (5 mm in diameter) were inserted onto PDA plate containing spread bacteria. Cultures were incubated for 7 days at 26°C and the diameter of the fungal mycelium was estimated. Pure cultures of each fungus were used as a control. Each experiment was performed in triplicate. The fungal growth inhibition zone was estimated by the following formula:


inhibition %=(C-B)/C ×100

where C is the diameter of fungi colony in the control plate, B is the diameter of fungi colony that grew in the presence of rhizobacteria (Wonglom et al., 2019).

## 3 Results

### 3.1 Assay of plant growth–promoting traits *in vitro*

The total rhizobacterial population was counted from different plant growth stages: the vegetative, flowering, and maturity. This analysis showed that the total bacterial load was the highest in the flowering stage followed by a maturity and vegetative stage (**Supplementary Table S1**). Fifty canola rhizospheric bacteria were isolated from the three plant growth stages and were tested for PGP traits including IAA, phosphate, ACC deaminase, siderophores, chitinases, HCN, and ammonia production. Ammonia and HCN production by bacterial strains can also have a positive effect on plant growth, for example by elongation of plant roots and shoots (Bhattacharyya et al., 2020). The plant growth promotion assay *in vitro* showed that rhizobacterial strains isolated from different growth stages exhibited various PGP traits (**Supplementary Figure S1**). Six isolates possessed the best PGP properties (**Table 1**). All of the strains were able to produce IAA, sequester siderophores and solubilize phosphates. Among these strains 2RO45 was the most effective regarding all three PGP. RO37 demonstrated the highest ACC deaminase activity. Three of the isolates: RO33, RO45, and 3RO30 were able to produce chitinases. Moreover, RO33 and 3RO30 strains were HCN and ammonia producers.

**Table 1:** Plant growth–promoting traits by selected PGPR strains.

Plant growth–promoting traits	Isolates					
	RO33	RO37	2RO24	2RO30	2RO45	3RO30
IAA (µg/mL)	19.59 ± 1.25^b^	16.62 ± 1.33^c^	17.85 ± 1.56^c^	22.57 ± 1.11^b^	25.84 ± 1.21^a^	13.04 ± 1.56^d^
P- solubilization (SoI)	1.0 ± 0.11^d^	3.0 ± 0.34^c^	3.0 ± 0.27^c^	8.0 ± 0.89^b^	10.0 ± 1.21^a^	2.0 ± 0.17^d^
ACC (nmol α-ketobutyrate/mg protein/h)	278.2 ± 13.5^c^	1846.6 ± 29.4_a_	115.8 ± 5.5^e^	178.7 ± 9.69^d^	1198.1 ± 23.6^b^	0.0^f^
Siderophore (d_halo_/d_colony_)	2.0 ± 0.19^c^	3.8 ± 0.50^b^	4.3 ± 0.64^a^	2.0 ± 0.15^c^	4.8 ± 0.66^a^	3.0 ± 0.38^b^
Chitinase (clear zone in mm)	1.0 ± 0.14^c^	0.0^d^	2.0 ± 0.18^a^	0.0^d^	0.0^d^	1.5 ± 0.15^b^
HCN	+	−	−	−	−	+
Ammonia	+	−	−	−	−	+

Values are mean ± SE of three replicates; (+) positive result; (−) negative result. Different letters indicate significant differences based on the Tuckey HSD test, p< 0.05.

### 3.2 Greenhouse experiment in sterile and non-steriled conditions

**Figure 1:**
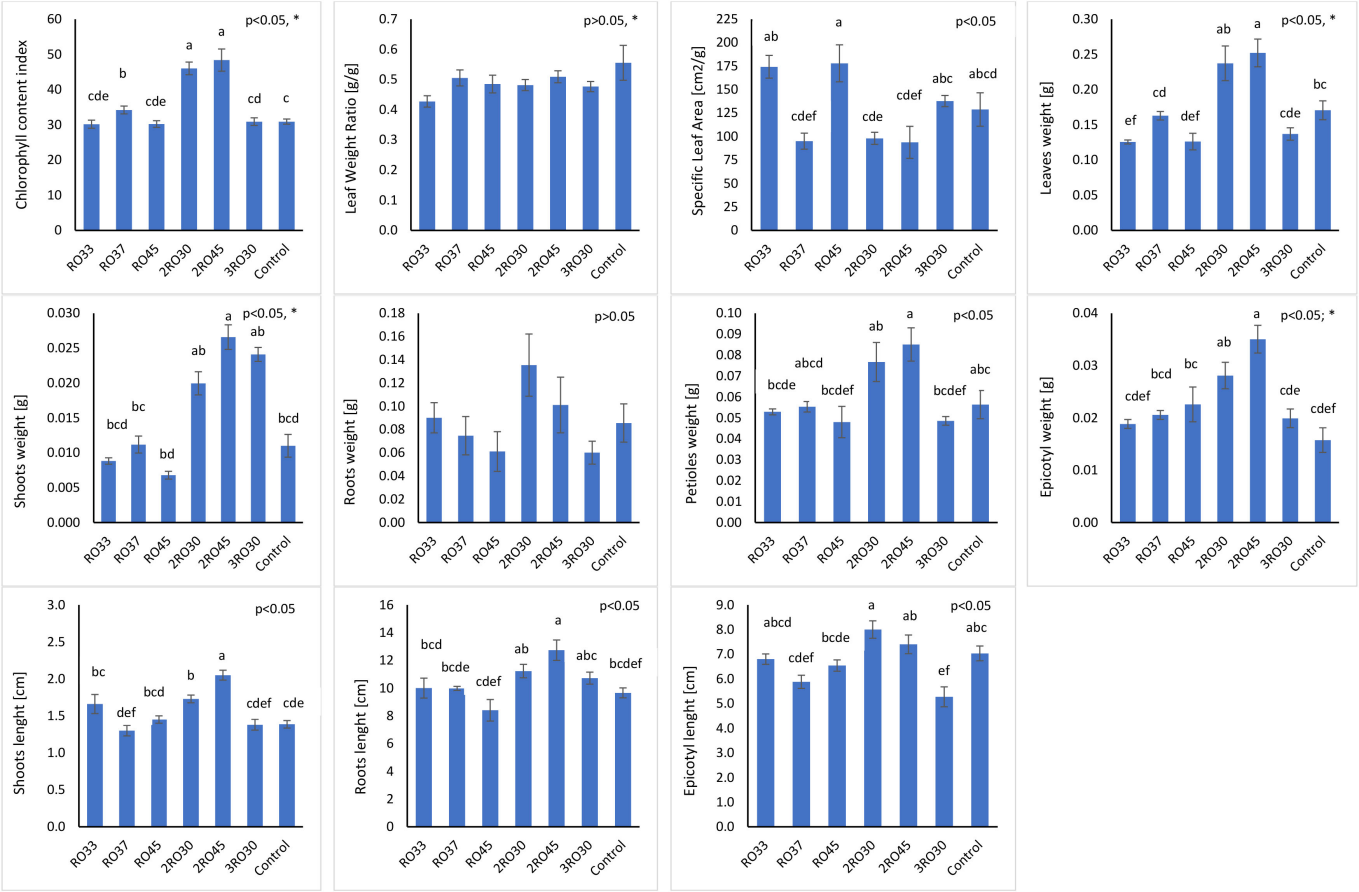
Plant growth parameters in sterile soil. Different letters indicate significant differences based on Tukey test as a *post hoc*, *p*< 0.05; *not normal distribution (test for equal medians: Mann–Whitney).

**Figure 2:**
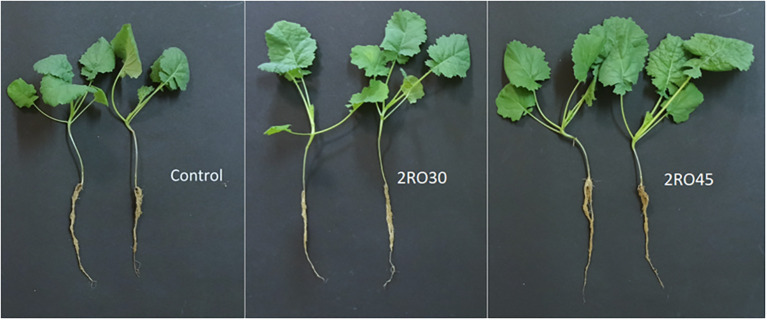
*Peribacillus frigoritolerans* 2RO30 and *Pseudomonas sivasensis* 2RO45 effect on canola growth compared to untreated control under sterile conditions.

Six bacterial strains identified as *Pseudomonas* sp. RO33, *Pseudomonas poae* RO37, *Pseudomonas kairouanensis* RO45, *Peribacillus frigoritolerans* 2RO30, *Pseudomonas sivasensis* 2RO45, and *Pseudomonas migulae* 3RO30 were used in greenhouse experiment to evaluate their *in-vivo* ability to stimulate canola growth under sterile conditions (**Figure 1**). The results showed that *P. frigoritolerans* 2RO30 and *P. sivasensis* 2RO45 significantly increased CCI and roots length compared to the non-inoculated control and to seedlings inoculated with RO33, RO37, RO45, and 3RO30. In addition, these isolates significantly improved shoots length and epicotyl weight compared to the untreated control. Seedlings inoculated with *P. frigoritolerans* 2RO30 showed significantly higher mean value of epicotyl length compared to RO37, RO45, and 3RO30, while for *P. sivasensis* 2RO45, this parameter was higher compared to RO37 and 3RO30 strains. Inoculated canola with 2RO30 and 2RO45 had significantly higher shoots weight compared to non-inoculated plants and those inoculated with RO33, RO37, and RO45 strains. Moreover, *P. sivasensis* 2RO45 induced leaves weight compared to the control and to RO33, RO37, RO45, and 3RO30 treatments while, for petioles weight, significant increase was observed compared to seedlings inoculated with RO33, RO45, and 3RO30, but not to the control. The PGP effect of *P. frigoritolerans* 2RO30 and *P. sivasensis* 2RO45 under sterile conditions is visible in the **Figure 2**.

**Figure 3:**
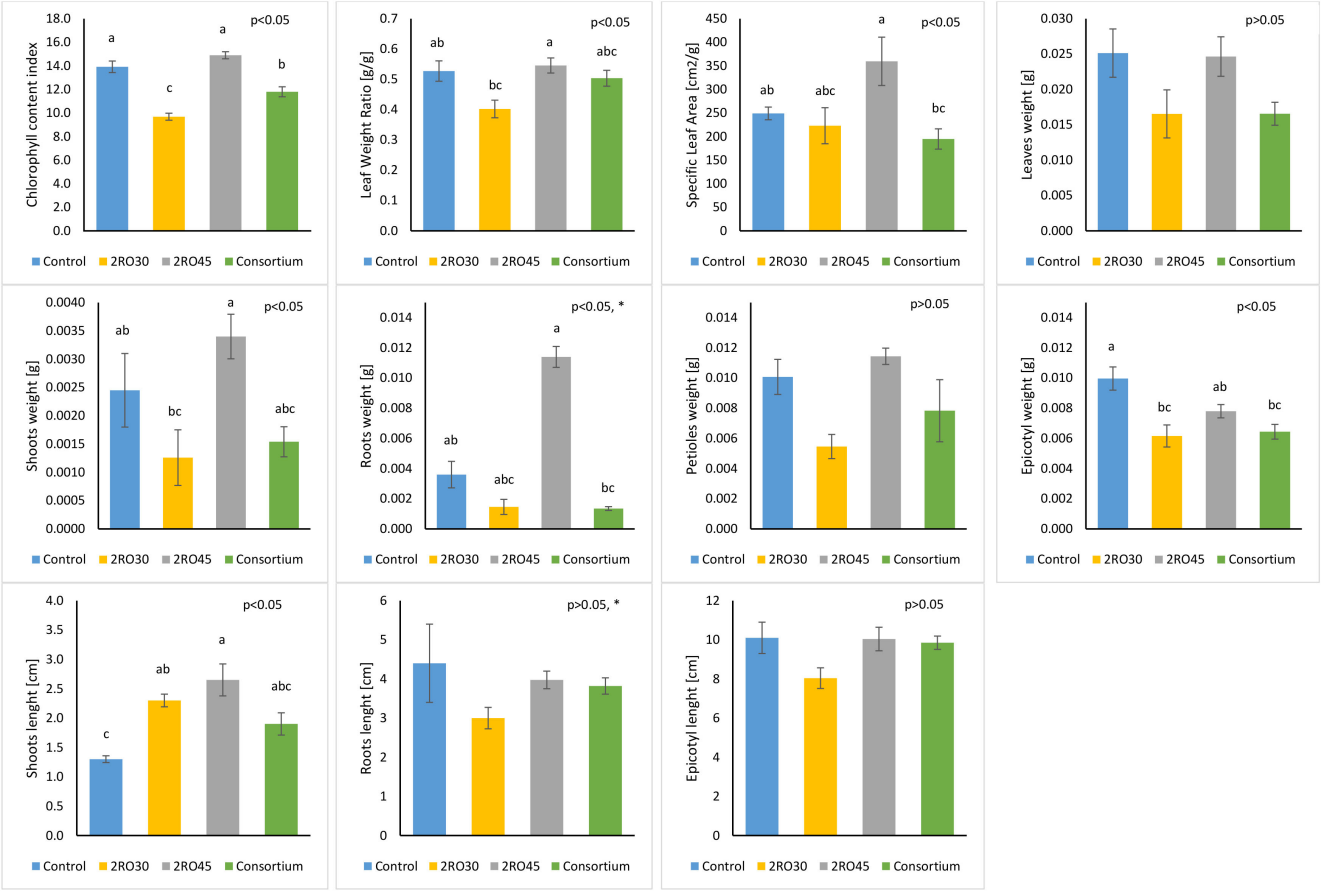
Plant growth parameters in non-sterile soil. Different letters indicate significant differences based on Tukey test as a *post hoc*, *p*< 0.05; *not normal distribution (test for equal medians: Mann–Whitney).

Based on growth promotion effects under sterile conditions *P. frigoritolerans* 2RO30 and *P. sivasensis* 2RO45 were selected for greenhouse experiment in non-sterile soil. Additionally, as compatibility between these strains was not observed, the consortium of the isolates was used for the experiment (**Figure 3**).

**Figure 4:**
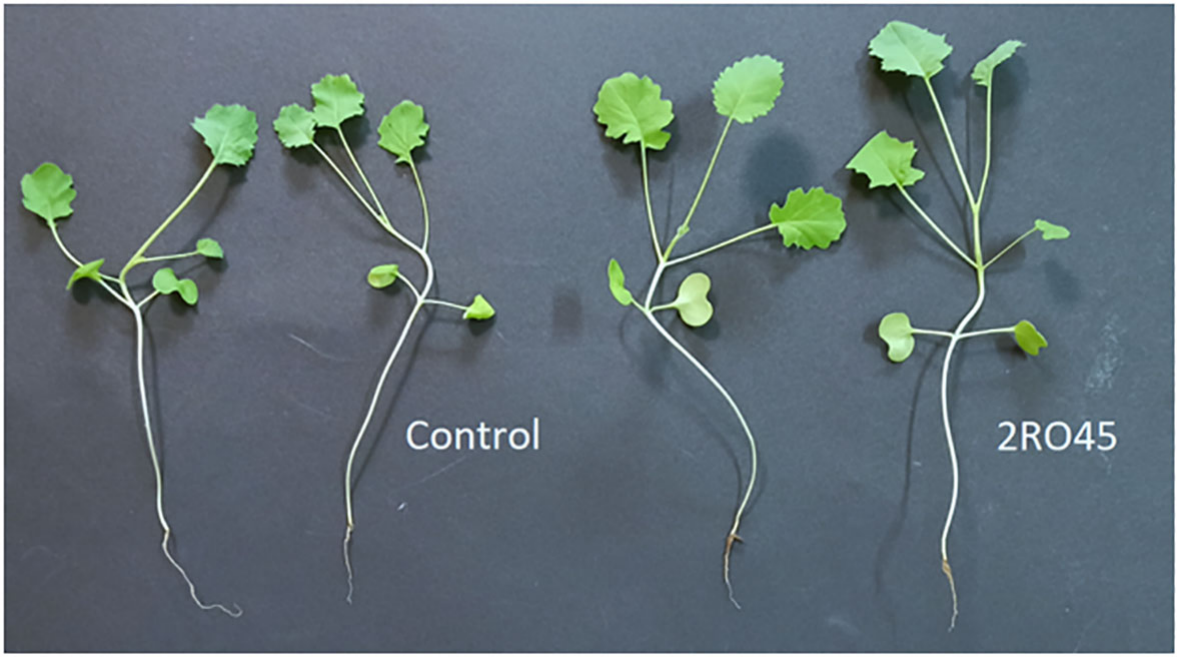
*Pseudomonas sivasensis* 2RO45 effect on canola growth under non-sterile conditions.

Our findings demonstrated that *P. sivasensis* 2RO45 significantly improved CCI compared to the untreated control and other inoculant treatments. Moreover, 2RO45 strain significantly increased shoots length compared to the non-inoculated control, but not to the other inoculated seedlings. Seedlings inoculated with the strain showed significantly higher SLA index and weight of roots compared to consortium but not to the control. Significant differences in LWR index and shoots weight between *P. frigoritolerans* 2RO30 and *P. sivasensis* 2RO45 were observed with higher mean values for *P. sivasensis* 2RO45. **Figure 4** showed the PGP effect of *P. sivasensis* 2RO45 compared to untreated control under non-sterile conditions.

The physicochemical analysis of the non-sterile soil (**Supplementary Table S2**) recorded potassium content of 350 mg/kg, followed by phosphorus content of 310 mg/kg, and magnesium content of 91 mg/kg. The ammonium nitrogen, nitrate nitrogen, and organic carbon contents were 6.57 mg/kg, 79.13 mg/kg, and 1.65%, respectively. The soil had a slightly acidic pH.

### 3.3 Genome sequencing, prediction of PGP genes, and biosynthetic gene clusters

Annotation of *P. frigoritolerans* 2RO30 and *P. sivasensis* 2RO45 genomes identified 5,435 and 5,797 coding genes, respectively, and 120 and 77 pseudogenes, respectively. In total, 1,877,684 and 2,803,949 reads were obtained from the whole-genome sequencing of 2RO30 and 2RO45 and assembled into 85 and 92 contigs with GC content of 40.19% and 59.63%, respectively (**Supplementary Table S3**).

**Figure 5:**
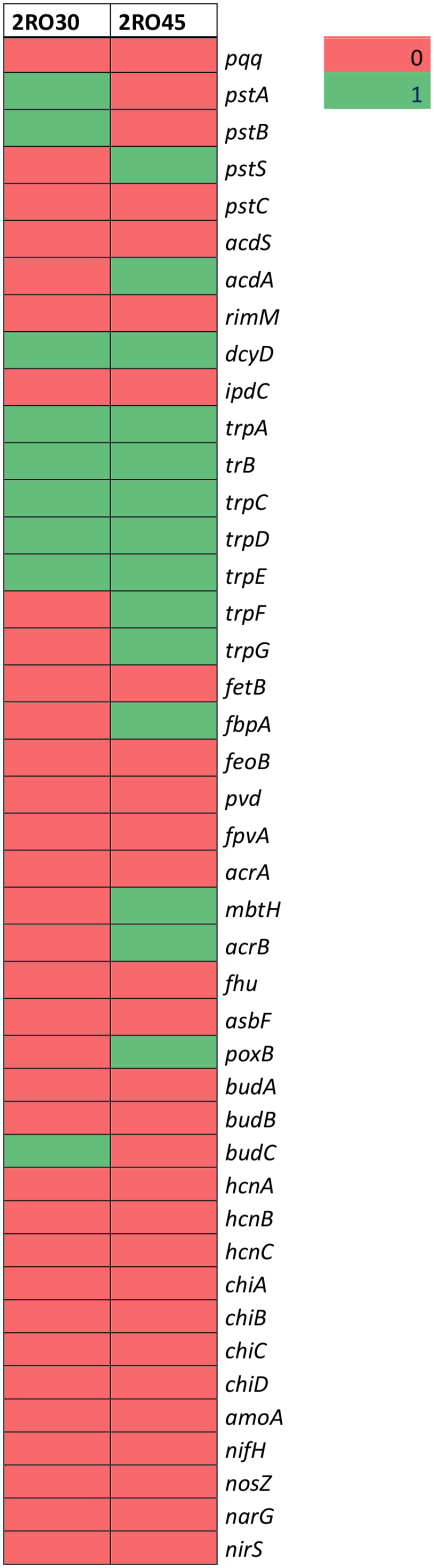
Genes responsible for plant growth–promoting characteristics.

Whole genome sequencing of the *P. frigoritolerans* 2RO30 and *P. sivasensis* 2RO45 revealed differences in identified PGP genes between strains (**Figure 5** and **Supplementary Table S4**). 2RO30 genome harbored *pstA*, and *pstB*, while 2RO45 genome coded for *pstS*, genes, which are responsible for phosphate transport. The genomes of 2RO30 and 2RO45 included genes involving in ACC deaminase (*dcyD*) and IAA (*trpA,B,C,D,E*) production. However, additional genes contributing to ACC deaminase (*acdA*) and IAA (*trpF, trpG*) were detected in *P. sivasensis* 2RO45 genome. Among 2RO30 and 2RO45 genomes, only 2RO45 genome contained genes involved in siderophores sequestration (*fbpA*, *mbtH*, and *acrB*). Moreover, genes related to acetoin and butanediol synthesis were found in 2RO30 and 2RO45 genomes. 2RO30 genome coded for *budC* gene, while 2RO45 coded for *poxB.*

**Figure 6:**
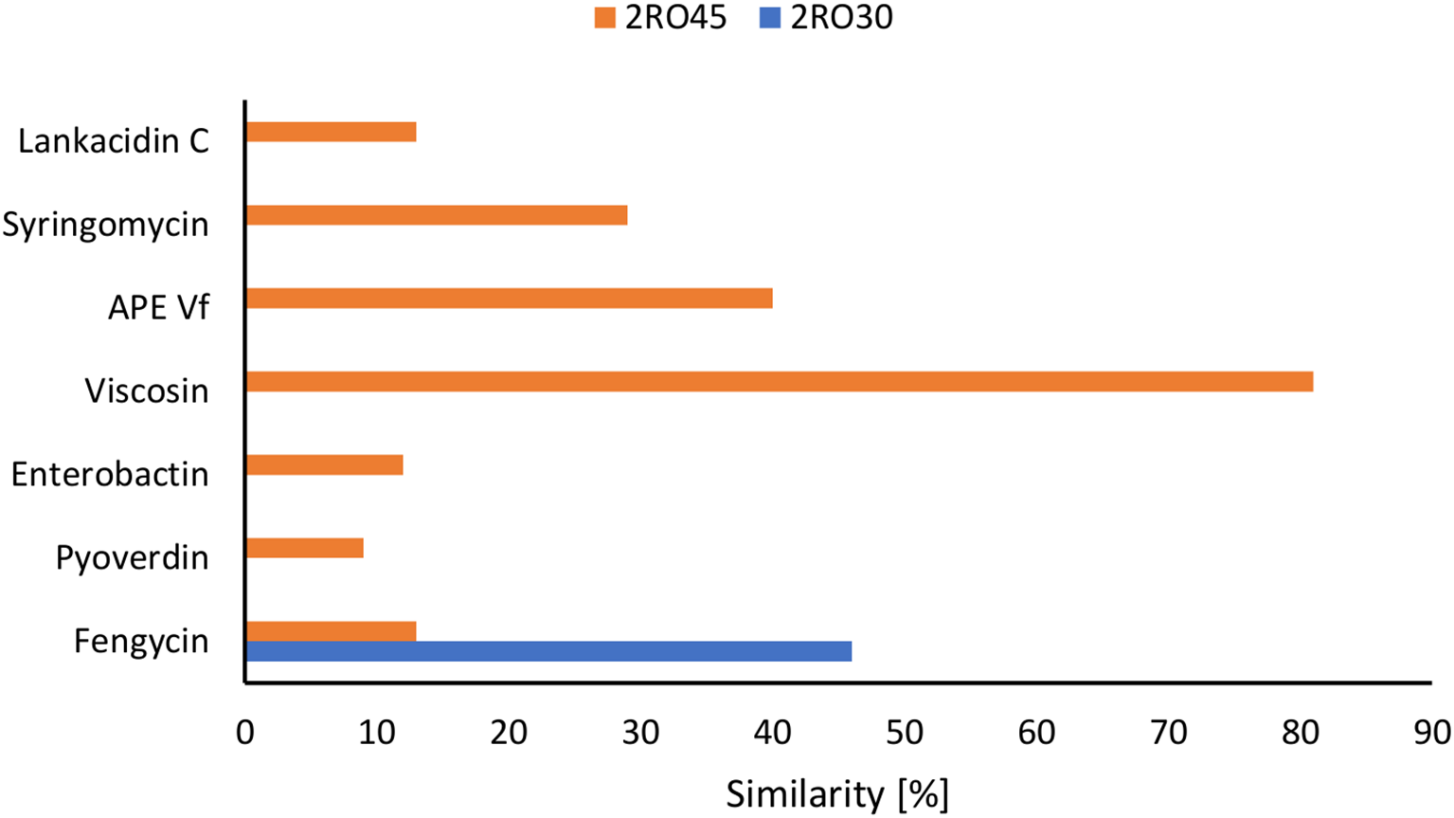
Detection of the secondary metabolite genes in *Peribacillus frigoritolerans* 2RO30 and *Pseudomonas sivasensis* 2RO45 genomes.

The analysis of biosynthetic gene clusters (BGC) revealed the presence of betalactone (fengycin) in both *P. frigoritolerans* 2RO30 and *P. sivasensis* 2RO45 genomes. Moreover, four additional type of genes, including nonribosomal peptide synthetases (NRPS) (viscosin, syringomycin, and pyoverdin), terpene (enterobactin), arylpolyene (APE Vf), and redox-cofactor (lankacidin C) were detected in 2RO45 genome. The abovementioned BGCs of *P. frigoritolerans* 2RO30 and *P. sivasensis* 2RO45 are present in the **Figure 6** and **Supplementary Table S5**.

### 3.4 Assay of plant pathogens growth inhibition *in vitro*

**Figure 7:**
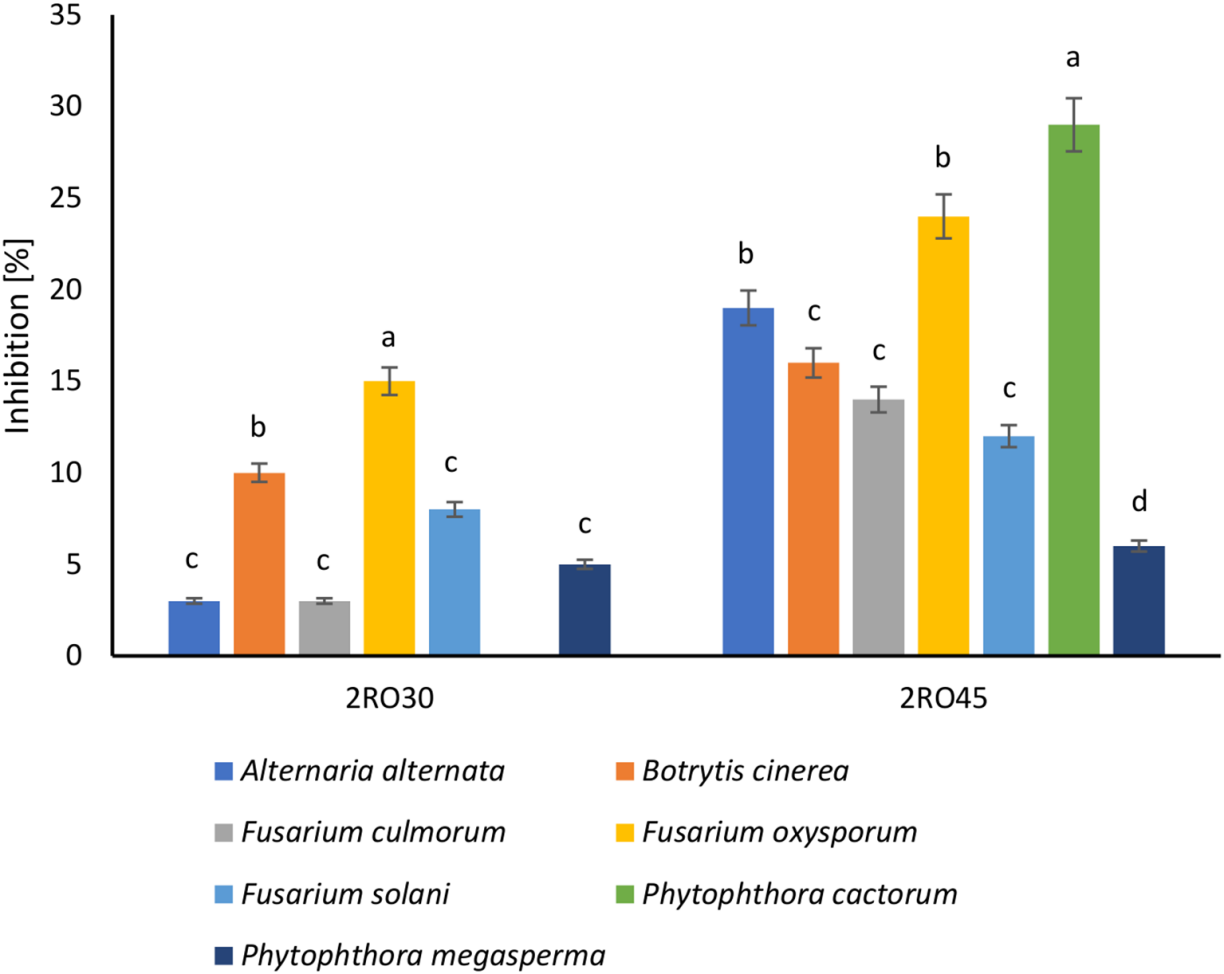
Inhibition of plant pathogens by *Peribacillus frigoritolerans* 2RO30 and *Pseudomonas sivasensis* 2RO45. Different letters indicate significant differences based on the Tuckey HSD test, *p*< 0.05.

The phytopathogens inhibition assay revealed that *P. sivasensis* 2RO45 had antifungal effect against all tested fungi. 2RO45 strain inhibited the mycelial growth of six plant pathogens (*A*. *alternata*, *B. cinerea*, *F*. *culmorum*, *F*. *oxysporum*, *F*. *solani*, and *P*. *cactorum*) with the inhibition rate of more than 10%. Whereas, *P. frigoritolerans* 2RO30 antagonistic effect in the rate greater than or equal to 10% was observed only against *B. cinerea* and *F. oxysporum* (**Figure 7**).

## 4 Discussion

PGPR can promote plant growth by direct and indirect mechanisms, which can be active simultaneously or independently at different plant growth stages (Kumar et al., 2012). In our study, the bacterial community was enumerated from three canola growth stages: vegetative, flowering and maturity. The results showed that the total bacterial load was the highest in the flowering stage followed by a maturity and vegetative stage. It can be explained by the fact that, when a plant grows, rhizospheric bacteria enter the plant system at vegetative stage and multiply during flowering stage but again start declining at maturity stage, for example, due to water stress, nutrient deficiency or free radical formation (Marag and Suman, 2018). Our results are in agreement with other studies. Marag and Suman (2018) enumerated endophytic population from different maize growth stages and found that the maximum bacterial loads were at the flowering stage. However, it is not known whether there is any association between the isolation of bacteria from different growth stages and their plant growth promoting (PGP) characteristics. Our study showed that rhizobacterial strains isolated from different periodic growth stages exhibited various PGP traits. However, a higher number of strains with the following PGP activities: IAA, ACC, phosphates, siderophores, and ammonia production were isolated from the flowering stage. This could be explained by much higher bacterial loads at the flowering stage than at the vegetative and maturity stages.

In addition, our results showed that two strains isolated from flowering stage growth: *P. frigoritolerans* 2RO30 and *P. sivasensis* 2RO45 were the most effective in stimulating canola growth in sterile conditions. According to Pérez-Montaño et al. (2014) when PGPR promote plant growth under sterile conditions, their effect on plant development should be evaluated also under non-sterile conditions. Therefore, to demonstrate the plant growth promotion ability of *P. frigoritolerans* 2RO30 and *P. sivasensis* 2RO45, a greenhouse experiment in non-sterile conditions was performed. In addition to testing the PGP effect of *P. frigoritolerans* 2RO30 and *P. sivasensis* 2RO45, a consortium of these two strains was used in the non-sterile experiment. Swiontek Brzezinska et al. (2022) showed that co-inoculation of three rhizobacterial strains from different taxa (*Pseudomonas*, *Sphingobacterium* and *Microbacterium*) enhanced canola growth in comparison to single-inoculant treatments. However, our results showed that canola growth parameters increased only when plants were inoculated with *P. sivasensis* 2RO45. Interestingly, *P. frigoritolerans* 2RO30, which promoted plant growth under sterile conditions, did not promote canola under non-sterile conditions. Beneficial effects of PGPR on plant development are highly variable under natural conditions due to the native rhizosphere microbial communities’ presence (Pacheco da Silva et al., 2022). According to Bhattacharyya et al. (2018), certain indigenous microbes might negatively affect the function and survival of introduced PGPR. Our results could be explained by that *P. frigoritolerans* 2RO30 failed in competition with soil natural microbiota and therefore it did not promote in non-sterile conditions. Moreover, it could be associated with differences in PGP characteristics *in vitro* and PGP genes between *P. frigoritolerans* 2RO30 and *P. sivasensis* 2RO45 strains.

High IAA production and phosphates solubilization features responsible for promoting canola growth under sterile conditions have been confirmed by the presence of appropriate genes in the genomes of these strains. However, *P. sivasensis* 2RO45 coded for many additional genes related to ACC and IAA production, such as *acdA* and *trpF,G*, which could have contributed to better canola growth promotion both in sterile and non-sterile soil. PGPR that produce IAA may increase root biomass, allowing the plant better access to soil nutrients and water uptake, which in turn can stimulate plant growth (Gilbert et al., 2018). Whereas, ACC deaminase can facilitate adaptation and survival of plants and increase roots length, thereby promote plant growth (del Carmen Orozco-Mosqueda et al., 2020). Świątczak et al. (2023c) found that the presence of *trpA,B,C,D,E,F,S* and *pqq, pstAB* genes in *Brevibacillus laterosporus* K75 genome and its high IAA and phosphates activity *in vitro* contributed to maize growth promotion under sterile and non-sterile conditions.

Apart from the genes mentioned above, 2RO45 genome contained *fbpA*, *mbtH*, *acrB* genes involved in siderophores production. BGC analysis revealed the presence of siderophore–enterobactin in its genome. Moreover, *P. sivasensis* 2RO45 showed the highest siderophores production in the PGP assay *in vitro*. It was reported that siderophores are responsible for plant growth promotion indirectly by inhibition of plant pathogens. However, siderophores can also stimulate plant species directly by enhancing iron (Fe) uptake in plants (dos Santos et al., 2020). Therefore, we assumed that high siderophores activity *in vitro* and the presence of many genes related to siderophores production in comparison to *P. frigoritolerans* 2RO30 are also responsible for *P. sivasensis* 2RO45 PGP effect under non-sterile conditions.

Moreover, *P. sivasensis* 2RO45 inhibited the mycelial growth of all fungi and with higher inhibition rates than *P. frigoritolerans* 2RO30. These observations are confirmed by genome mining and BGC analysis where *P. sivasensis* 2RO45 harbored six more secondary metabolite cluster genes than *P. frigoritolerans* 2RO30. The founded NRPS: syringomycin, pyoverdine, and viscosin are well known as antifungal agents (Bensaci et al., 2011; Alsohim et al., 2014; Sass et al., 2021). Fengycin is a lipopeptide producing by *Bacillus amyloliquefaciens* PPL, which contributes to antifungal activity against *Fusarium oxysporum* (Kang et al., 2020). Arylpolyene from *Vibrio fischeri* ES114 (APE Vf) was found in many *Pseudomonas* species (Gaete et al., 2022; Han et al., 2022) and according to Dutta et al. (2020) this lipid influences the antifungal effect of *P. fluorescens* NBC275. Strain *Streptomyces rochei* FS18 producing polyketide antibiotic–lankacidin and its derivatives, showed significant antifungal effect against *Aspergillus niger* (Cao et al., 2015). It can be assumed that these BGCs were responsible for the antifungal activity of *P. sivasensis* 2RO45 against plant pathogens, and if it has more possibilities of defense against pathogens, it promotes plants more effectively.

## 5 Conclusions

*Pseudomonas sivasensis* 2RO45 and *Peribacillus frigoritolerans* 2RO30 possessing the highest IAA production and phosphates solubilization, showed the best promoting effect on canola growth under sterile conditions. Under non-sterile conditions, only *Pseudomonas sivasensis* 2RO45 promoted canola growth, which can be associated with the presence of additional genes responsible for ACC deaminase (*acdA*), IAA (*trpF, trpG*), and siderophores production (*fbpA*, *mbtH*, and *acrB*) in its genome. Our study is the first report describing *Pseudomonas sivasensis* 2RO45 as a plant growth promoter, both under controlled and natural conditions, thus suggesting its application in improving canola yield, by improving nutrient availability, enhancing stress tolerance, and reducing the environmental impact of farming practices. Its application aligns with the principles of environmentally friendly and economically viable agriculture. However, more research about how this strain competes with native soil microbes is necessary.

## Data availability statement

The datasets presented in this study can be found in online repositories. The names of the repository/repositories and accession number(s) can be found in the article/**Supplementary Material**.

## Author contributions

JŚ wrote the original draft, made plant growth – promoting assays *in vitro* and *in vivo*, conducted statistical data and genome analysis, and prepared the manuscript editorially; AK coordinated the study, and checked the validity of the original draft; MS designed and coordinated the study, checked the validity of the original draft. All authors contributed to the article and approved the submitted version.
