# Supplementary material for: Plant growth–promoting rhizobacteria: Peribacillus frigoritolerans 2RO30 and Pseudomonas sivasensis 2RO45 for their effect on canola growth under controlled as well as natural conditions
Source: Front Plant Sci. 2024 Jan 8;14:1233237. doi: 10.3389/fpls.2023.1233237 (PMC10800854; doi:10.3389/fpls.2023.1233237)
Supplement: Supplementary file 1 [file DataSheet_1.docx]

**Figure S1** Number of stains showing PGP properties isolated from different plant growth stages: vegetative (RO), flowering (2RO) and maturity (3RO)

**Table S1** Total rhizobacterial load from different canola growth stages

| **Plant growth stage** | **Total bacterial load (x 10^3^ CFU/mL)** |
| --- | --- |
| Vegetative | 9.8 |
| Flowering | 12.3 |
| Maturity | 11.1 |

**Table S2** The physical and chemical analysis of the non-sterile soil

| **Parameter** | **Result** |
| --- | --- |
| pH | 6.5 |
| Phosphorus [mg/kg] | 310.0 |
| Potassium [mg/kg] | 350.0 |
| Magnesium [mg/kg] | 91 |
| Ammonium nitrogen [mg/kg] | 6.57 |
| Nitrate nitrogen [mg/kg] | 79.13 |
| Organic carbon [%] | 1.65 |

**Table S3** Genomes assemblies and annotations

|  | ***Peribacillus frigoritolerans* 2RO30** | ***Pseudomonas sivasensis* 2RO45** |
| --- | --- | --- |
| Bioproject | PRJNA876229 | PRJNA876229 |
| Biosample | SAMN30648207 | SAMN30648208 |
| Assembly | GCA_025209795.1 | GCA_025209875.1 |
| Level | Contig | Contig |
| GC [%] | 40.19 | 59.63 |
| WGS | JAOAQM01 | JAOAQN01 |
| Genes | 5,435 | 5,797 |
| Pseudogenes | 120 | 77 |
| Number of reads | 1,877,684 | 2,803,949 |
| Total sequence length | 5,517,502 | 6,309,106 |
| Number of contigs | 85 | 92 |
| Contig N50 | 2,937,248 | 460,348 |
| Contig L50 | 1 | 5 |

**Table S4** Genes responsible for plant growth – promoting characteristics

| **Pathway** | **2RO30** | **2RO45** | **Genes** |
| --- | --- | --- | --- |
| Degradation of  phosphonates;  phosphate transport | - | - | *pqq* |
|  | + | - | *pstA, pstB* |
|  | - | + | *pstS* |
|  | - | - | *pstC* |
| ACC deaminase production | - | - | *acdS* |
|  | - | + | *acdA* |
|  | - | - | *rimM* |
|  | + | + | *dcyD* |
| IAA production;  L-tryptophan production | - | - | *ipdC* |
|  | + | + | *trpE, trpA, trpD, trpC, trpB* |
|  | - | + | *trpF, trpG* |
| Siderophore transport | - | - | *fetB* |
|  | - | + | *fbpA* |
|  | - | - | *feoB* |
|  | - | - | *pvd, fpvA, acrA* |
|  | - | + | *mbtH* |
|  | - | + | *acrB* |
|  | - | - | *fhu* |
|  | - | - | *asbF* |
| Acetoin and butanediol synthesis | - | + | *poxB* |
|  | - | - | *budA, budB* |
|  | + | - | *budC* |
| HCN production | - | - | *hcnA, hcnC, hcnB* |
| Chitinase production | - | - | *chiA, chiB, chiC, chiD* |
| Nitrogen cycle | - | - | *amoA, nifH, nosZ, narG, nirS* |

**Table S5** Biosynthetic cluster genes in *Peribacillus frigoritolerans* 2RO30 and *Pseudomonas sivasensis* 2RO45 genomes

| **Region** | **From** | **To** | **Type** | **Most similar gene cluster** | **Similarity [%]** |
| --- | --- | --- | --- | --- | --- |
| ***Peribacillus frigoritolerans* 2RO30** | | | | | |
| Region 1.2 | 2,130,359 | 2,154,527 | betalactone | fengycin | 46 |
| ***Pseudomonas sivasensis* 2RO45** | | | | | |
| Region 1.1 | 276,750 | 329,661 | NRPS | pyoverdin | 9 |
| Region 1.2 | 335,507 | 357,081 | terpene | enterobactin | 12 |
| Region 5.1 | 328,232 | 441,499 | NRPS | viscosin | 81 |
| Region 6.1 | 282,883 | 326,458 | arylpolyene | APE Vf | 40 |
| Region 9.1 | 34,055 | 62,398 | betalactone | fengycin | 13 |
| Region 9.2 | 181,413 | 211,716 | NRPS | syringomycin | 29 |
| Region 12.1 | 45,779 | 67,926 | redox-cofactor | lankacidin C | 13 |
